# Green synthesis of CuO nanoparticles using thyme extract and their application as cephalexin carriers against *Klebsiella pneumoniae*

**DOI:** 10.1039/d5na00726g

**Published:** 2025-11-11

**Authors:** Luma Dali, Sumood Al-Hadithy, Aws Z. Abdulmajeed, Alauddin M. Mahdi, M. A. Hamed

**Affiliations:** a University of Anbar, College of Basic Education-Hadeetha Anbar Iraq awsa.zabin@uoanbar.edu.iq; b Tikrit University, College of Pharmacy Tikrit Iraq

## Abstract

Cephalexin is commonly used to treat various bacterial infections. Given the escalating issue of antibiotic resistance worldwide and the need for alternative treatment methods, this study focused on enhancing the antimicrobial efficacy of cephalexin against pneumonia, which remains a major cause of mortality among children. *Klebsiella pneumoniae* strains were isolated from pneumonia patients. Wild thyme extract was used in the synthesis of environmentally friendly copper oxide nanoparticles, which were characterized using advanced analytical techniques. Cephalexin was loaded onto these nanoparticles to evaluate their enhanced antibacterial efficacy against *Klebsiella pneumoniae*. In addition, comprehensive studies were conducted on the antioxidant properties and anti-biofilm capabilities of the newly synthesized cephalexin–CuO nanocomposite. UV-visible spectroscopy, X-ray diffraction, and magnetic resonance confirmed the successful binding of cephalexin to the synthesized nanoparticles. The bioactivity and antioxidant evaluations also revealed promising results. The antibacterial activity test showed that the copper nanoparticles exhibited a minimum inhibitory concentration (MIC) in the range of 1.8–2.2 mg ml^−1^, indicating enhanced efficacy of the nanocomposite. Moreover, the cephalexin–copper oxide nanocomposite achieved complete bacterial inhibition at an optimal concentration of 30 mg ml^−1^, while biofilm formation inhibition tests identified 20 mg ml^−1^ as the optimal concentration for suppression of pulmonary biofilm generation by *Klebsiella pneumoniae*. These findings underscore the potential of the cephalexin–CuO nanocomposite as a promising candidate for the development of innovative antibacterial and antioxidant solutions.

## Abstract 2

This study aims to improve the biological properties of cefalexin by using green synthesized copper oxide nanoparticles against *Klebsiella* bacteria. The hybrid compound achieved complete inhibition of the bacteria.

## Graphical abstract

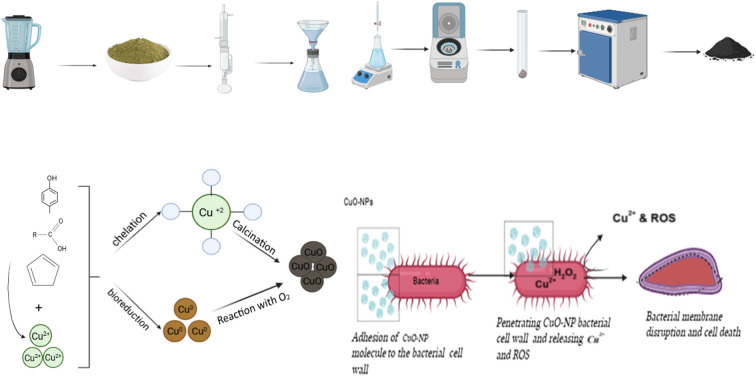

## 1. Introduction

Infectious pneumonia (IPN) is the most common disease among newborns and a leading cause of death from infectious diseases.^1,2^ IPN has a high incidence and rapid progression, accounting for 14% of all deaths in children under five. Furthermore, IPN is responsible for over 25% of all newborn fatalities.^3,4^ The overall rate of pneumonia remains high, and further research is needed to improve care for at-risk populations, such as the elderly.^5^

The Enterobacteriaceae family's Gram-negative bacterium*, Klebsiella pneumoniae*, is a significant opportunistic pathogen causing severe pneumonia and other systemic illnesses.^6^ It can be classified into two types: classic *Klebsiella pneumoniae* (cKP), which ranks second only to *E. coli* in infection rates, and hypervirulent *Klebsiella pneumoniae* (hvKP). They lead to severe pneumonia and a variety of other infections, including respiratory tract infections, urinary tract infections, blood poisoning, and liver diseases.^7^ Pneumococcal infections can become chronic in some cases because they form a biofilm, which acts as a protective barrier against the host's immune system and antibiotics.^8,9^ Multidrug-resistant (MDR) and hypervirulent strains have emerged in recent years, posing a major threat to global health. New therapeutic strategies are urgently needed, especially given *K. pneumonia's* growing resistance to β-lactam antibiotics, such as cephalosporins and even carbapenems.^10–12^

Cephalexin is a first-generation β-lactam antibiotic from the oral cephalosporin group used to treat a wide variety of infections. It is suitable for both adults and children.^13^ Cephalexin is known for its effectiveness against many bacteria, particularly making it a valuable antibiotic in medical treatment.^14,15^ It is classified as a Class I drug under the Bioequivalence Classification System (BCS), which indicates high solubility and permeability.^16^ Additionally, cephalexin has shown activity against human liver cancer cells (HepG2) when loaded onto chitosan nanoparticles, representing a potential new application beyond its traditional role in fighting bacterial infections.^17,18^ Its chemical formula is C_16_H_17_N_3_O_4_S, and its molecular weight is 365.4 g mol^−1^. It is affordable and relatively safe. These factors contribute to cephalexin's status as a common first-line treatment for infections.^16^ However, its clinical efficacy has been restricted because of growing resistance among Gram-negative bacteria. Potential answers to these problems can be found in nanotechnology. The FDA considers metal oxide nanoparticles, especially copper oxide nanoparticles (CuO-NPs), to be safe materials because of their intrinsic antibacterial, antioxidant, and biocompatible qualities.^19–21^ Due to the limitations of the chemical and physical preparation of nanomaterials, the green synthesis method uses plant extracts as reducing agents to form nanoparticles. This method can overcome these challenges and promote environmentally friendly chemistry.^22–25^ Thyme (*Thymus* spp.), belonging to the Lamiaceae family, is a good option for the synthesis of green nanoparticles because it includes bioactive phytochemicals with potent antibacterial and antifungal properties.^26,27^ Thyme is also an effective treatment for chronic fungal diseases, chest infections, asthma, respiratory infections, and whooping cough.^28^ This study took advantage of these characteristics by creating CuO-NPs using thyme extract and then loading them with cephalexin. Little research has examined the combination of CuO-NPs with cephalexin as a green synthesis-prepared nanocomposite, despite the fact that both substances independently have antibacterial activity. Specifically, nothing is known about how cephalexin-loaded CuO-NPs can fight MDR *K. pneumoniae*, reduce oxidative stress, and prevent the formation of biofilms. By synthesizing CuO-NPs with thyme extract, describing their physicochemical characteristics, and assessing the antibacterial, antioxidant, and anti-biofilm effects of cephalexin-loaded CuO-NPs, this study seeks to close this gap.

## 2. Methodology

### 2.1. Plant collection

Wild thyme was sourced from the arid region of Al-Haqlaneyah, located in Haditha District, Anbar Province, Iraq (34°05′21′′N 42°21′16′′E). The collected plants were carefully cleaned with water to remove any surface contaminants and subsequently air-dried to prepare them for the extraction process.

### 2.2. Preparation of thyme extract

A total of 25 grams of thyme leaves underwent a meticulous cleaning process using distilled water to remove impurities. Following this, the leaves were mechanically ground to produce a fine powder. The resulting powder was then placed in a Soxhlet apparatus for the extraction of bioactive compounds, utilizing 100 ml of methanol (70%) at a temperature of 60 °C. Once the extraction process was complete, the sample was cooled, filtered, and transferred into opaque containers. It was stored at a controlled temperature of 4 °C for less than one week to ensure its readiness for application in the synthesis of copper oxide nanoparticles.
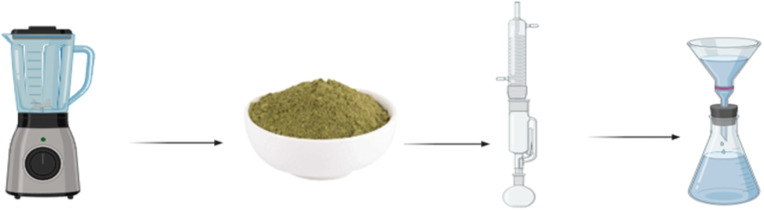


### 2.3. Green synthesis of copper oxide nanoparticles (CuO)

A 90 ml solution of copper chloride (100 mM) was prepared using deionized water and thoroughly homogenized using a magnetic stirrer. Subsequently, 10 ml of thyme leaf extract was introduced dropwise *via* a pipette over a period of 20 minutes, with continuous stirring at room temperature. During this process, a color change was observed, suggesting the formation of nanoparticles. Sodium hydroxide (0.1 M) was then added to adjust the pH to 14. The temperature was increased to 60 °C, and the solution continued stirring for two hours. Copper oxide nanoparticles were isolated by centrifugation at 3500 rpm for 30 minutes. The resulting precipitate was washed with deionized water to remove impurities and underwent an additional centrifugation step under the same conditions. The purified precipitate was left to dry in ambient air overnight and subsequently calcined at 250 °C using a combustion furnace. The final product was utilized for subsequent characterization and biological applications.
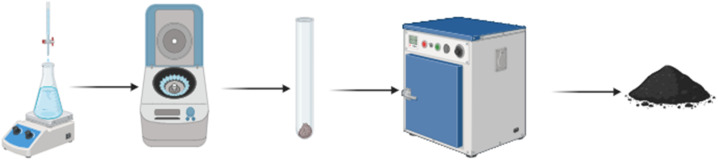


### 2.4. Identification of copper oxide nanoparticles

The composition of copper oxide nanoparticles was validated using an array of analytical methods. A UV-visible spectrophotometer (KLab alpha – USA) and FT-IR (Shimadzu) were employed to measure spectral properties. Their crystalline structure was examined through X-ray diffraction (XRD) analysis (using Gonio ASCII-2Theta-Intensity, wavelength λ = 1.54060Å), which also facilitated nanoparticle size estimation *via* the Debye–Scherrer equation.^29^ Additionally, a scanning electron microscope (SEM) provided detailed insights into the shape, size, and purity of the synthesized nanoparticles.

### 2.5. Bacterial isolation

In the microbiology laboratory at Haditha General Hospital in Iraq's Anbar Governorate, *Klebsiella pneumoniae* bacteria were isolated from patients suffering from pneumonia infections. Sputum samples were collected using swabs and cultured on blood agar and MacConkey agar using the streaking method. The cultures were then incubated at 37 °C for 24 hours to facilitate bacterial identification.

### 2.6. Bacterial diagnosis

After the bacterial growth was observed, *Klebsiella pneumoniae* was identified morphologically based on the shape and color of the colonies on MacConkey agar and blood agar. The diagnosis was further supported using biochemical tests and Gram staining. For confirmation, the Vitek-2 system was employed.

### 2.7. Cephalexin susceptibility test

Three approaches have been used in this research to test and confirm the susceptibility of cephalexin to *Klebsiella pneumonia*. The disk diffusion method (Kirby–Bauer method) was used as described by Bauer *et al.*^30^ and following the recommendations of the Clinical and Laboratory Standards Institute (2020).^31^ Discs of cephalexin were placed on Mueller Hinton agar containing *Klebsiella pneumonia* bacteria, and after incubation for 24 hours at 37 °C, the diameter of inhibition was measured.

The second method, as described by Alnuaimi *et al.* (2023),^32^ used different concentrations of cephalexin powder obtained from the Samarra Pharmaceutical Laboratory in Iraq. A bacterial suspension of *Klebsiella pneumoniae* was prepared at a concentration of 1.5 × 10^8^ colony-forming units per ml according to the 0.5 McFarland scale. 1 ml of the bacterial suspension was taken and cultured on Mueller–Hinton agar using the spreading-plate method. Different concentrations of cephalexin powder suspension were used (10–40 mg ml^−1^); 1 ml of each concentration was placed on a medium that contained *Klebsiella* bacteria. Two replicates were used for each concentration and incubated for 24 hours at 37 °C.

To further confirm the antibiotic sensitivity test, the Vitek-2 system device was also used to test whether the bacteria were sensitive, resistant, or resistant to the antibiotic.

All these methods have been performed to determine whether the bacteria were resistant (*R*), sensitive (*S*), or intermediately resistant (*I*) to cephalexin.

### 2.8. Antibacterial activity assay of CuO nanoparticles and MIC determination

Three concentrations of aqueous CuO-NPs were prepared to test their effectiveness against *Klebsiella pneumoniae* bacteria. A bacterial suspension was prepared at a concentration of 1.5 × 10^8^ colony-forming units per ml according to the 0.5 McFarland scale. The test was conducted according to the Alnuaimi *et al.* (2023) method;^32^ 1 ml of the bacterial suspension was added to plates containing Mueller–Hinton agar. The extract concentrations were then added successively, and two replications were made for each concentration. The plates were incubated at 37 °C for 24 hours to observe the effectiveness of aqueous CuO-NPs in inhibiting bacterial growth and to determine the minimum inhibitory concentration (MIC).

### 2.9. Loading cephalexin onto copper oxide nanoparticles

Drug loading represents a promising strategy for producing highly stable amorphous drugs with enhanced solubility, dissolvability, and bioavailability. In this context, the preparation of cephalexin-loaded CuO nanoparticles follows a systematic approach aimed at optimizing the antibacterial efficacy of the drug. This process is based on the methodology described by Herdiana *et al.*,^33^ with modifications introduced to refine certain aspects. To begin, a 2% w/v solution was prepared by dissolving 1 g of cephalexin in 50 ml of deionized water. Following this, 0.5 g of copper oxide nanoparticles was gradually added to the solution. For improved nanoparticle dispersion and homogeneous distribution, the mixture was exposed to ultrasonic treatment for 20 minutes at 20 °C, a crucial step for ensuring effective drug loading onto the nanoparticle surfaces. Careful temperature monitoring during ultrasonic treatment was imperative to prevent the decomposition of cephalexin or copper oxide nanoparticles. Subsequently, the solution was continuously stirred on a magnetic stirrer for 24 hours. This prolonged stirring period allowed cephalexin molecules ample time to bind effectively to the surface of the CuO nanoparticles while maintaining a homogeneous mixture—a critical factor for efficient drug incorporation. The resulting precipitate was then collected *via* centrifugation at 3500 rpm for 30 minutes and thoroughly washed with deionized water to eliminate any residual unreacted drug. Finally, the precipitate was left to air-dry overnight at room temperature. Validation of drug loading on copper oxide nanoparticles was conducted using advanced analytical techniques, including UV-visible spectroscopy (KLab alpha-USA), X-ray diffraction (XRD), and scanning electron microscopy (SEM), to confirm the successful incorporation of cephalexin onto the nanoparticle surfaces.

### 2.10. Antibacterial activity assay of the cephalexin–CuO composite

After preparing cephalexin–CuO-NPs, a test was conducted to determine its biological effectiveness in improving the antibiotic to inhibit the bacterial growth, where four concentrations of the compound were prepared (10–40 mg ml^−1^), also following the Alnuaimi *et al.* method in conducting this test.^32^ The bacterial suspension was prepared at a concentration of 1.5 × 10^8^ colony-forming units per ml according to the 0.5 McFarland scale and cultured it on Petri dishes containing Mueller–Hinton agar, and then added the concentrations of cephalexin–CuO-NPs; two replications were made for each concentration. It was incubated for 24 hours at 37 °C to observe whether the compound was effective in inhibiting bacterial growth or not, and to determine the concentration at which inhibition occurred if bacterial growth was inhibited.

### 2.11. Antioxidant activity (2,2-diphenyl-1-picrylhydrazyl radical scavenging assay)

The antioxidant activity of the cephalexin–CuO nanocomposite was evaluated by assessing its DPPH radical scavenging potential, following the methodology outlined by Takatsuka *et al.*^34^ For the analysis, a range of cephalexin–CuO nanocomposite concentrations (10–70 mg ml^−1^) was prepared in deionized water. Subsequently, 200 μL from each concentration was combined with 1 ml of a 0.2 mM 2,2-diphenyl-1-picrylhydrazyl (DPPH) solution in Eppendorf tubes. The mixtures were thoroughly vortexed and incubated in the dark. The absorbance for each sample was recorded at 517 nm, and the antioxidant activity was calculated using eqn (1).1
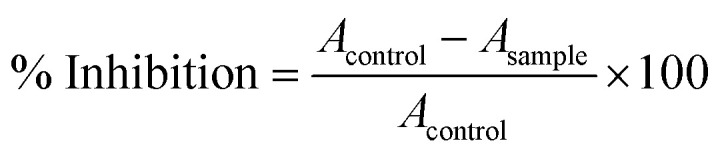
where *A* is the absorbance of the solutions after 30 min of reaction.

### 2.12. Biofilm inhibition assay

The method described by Ibne Shoukani *et al.* (2024)^35^ was used, whereby a series of dilutions (10–40 mg ml^−1^) of cephalexin–CuO nanocomposite solution was prepared using water as a solvent. Then, 200 microliters of each solution were added to a microtiter plate, followed by the positive control solution (broth and bacterial inoculum). Next, 10 microliters of bacterial inoculum were added to all wells except the negative control wells. The plate was incubated for 6 hours and then washed several times with water. 100 microliters of aqueous solution (1%) of crystal violet dye were added to all wells. After 15 minutes, the plate was washed several times and dried thoroughly. Then, 33% acetic acid was added to each well, and the absorbance was measured at 595 nm, where the color intensity indicates the presence of biofilm inhibition.

The decrease in the percentage of biofilms was determined using the following formula:^36^
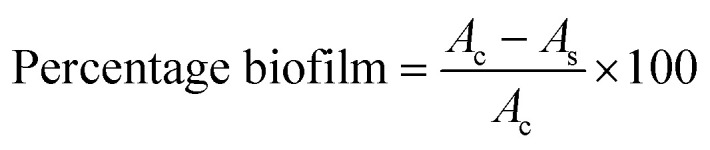
where *A*_c_ = OD_595_ of the positive control wells, and *A*_s_ = OD_595_ of cephalexin–CuO nanocomposite-treated wells (Shao *et al.* 2015).^36^

### 2.13. Cytotoxicity test

The MTT assay was used to demonstrate the cytotoxicity of the produced CuO-NPs using Baby Hamster Kidney (BHK-21) cell lines.^35^ In 96-well plates, cells were seeded at a density of 1 × 10^4^ cells per well. They were then incubated for the entire night at 37 °C in a humidified environment with 5% CO_2_. After that, the cells were exposed to varying doses of CuO-NPs (10, 20, 30, and 40 μg ml^−1^) for a duration of 24 hours. Doxorubicin served as the positive control, whereas untreated cells served as the negative control. Ten microliters of a 5 mg ml^−1^ MTT solution were added to each well after incubation, and the wells were then incubated for an additional four hours. A microplate reader was used to detect the absorbance at 570 nm after the formazan crystals were dissolved in 100 μL of DMSO. Cell viability was computed as a percentage.

Doxorubicin served as the positive control, whereas untreated cells served as the negative control. After incubation, 10 μL of MTT solution (5 mg ml^−1^) was added to each well, and the wells were incubated for a further 4 hours. A microplate reader was used to measure the absorbance at 570 nm following the dissolution of the resultant formazan crystals in 100 μL of DMSO. The following formula was used to calculate cell viability (%):
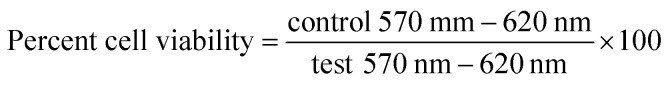


This test clarified the range of doses that demonstrate antibacterial action against *Klebsiella pneumoniae* while maintaining good biocompatibility with mammalian cells, hence illuminating the safety profile of CuO-NPs.

## 3. Results and discussion

Numerous plants and their extracts have been effectively utilized for synthesizing metal nanoparticles, demonstrating significant potential in biomedical applications. To the best of our knowledge, this study represents the first instance of biosynthesizing CuO-NPs using thyme extract—an approach that is both eco-friendly and cost-effective, without involving any harmful substances.

### 3.1. Ultraviolet-visible and FTIR spectra

**Fig. 1:**
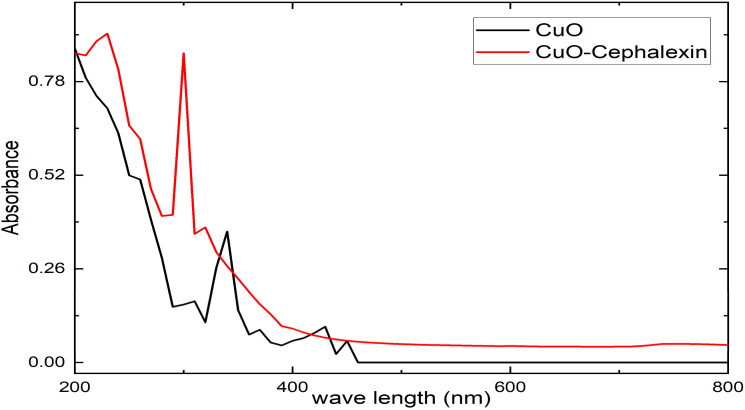
Ultraviolet-visible spectra of prepared copper oxide nanoparticles and cephalexin loaded on copper oxide nanoparticles.

In the present study, CuO-NPs were synthesized using wild thyme extract, and cephalexin was loaded onto the prepared nanoparticles. The particles were characterized using various diagnostic techniques, and the UV-vis absorption spectra of CuO-NPs were recorded using a spectrophotometer (KLAB-Alpha) in the range of 200–800 nm. It can be concluded from Fig. 1 that CuO-NPs do not show any clear absorption in the region where the wavelength exceeds 400 nm, while the highest absorption peak *λ*_max_ was at 340 nm, which can be attributed to the surface plasmon resonance (SPR) absorption range of CuO-NPs, which is consistent with the results of a previous study.^37^ Another study found two peaks at 285 nm and 340 nm corresponding to the SPR absorption range of the formed copper oxide nanoparticles. This SPR peak is a characteristic feature of nanoparticle structures and confirms the successful synthesis of copper oxide nanoparticles in the study,^38^ while Al-Fa'ouri indicated that the absorption peak for CuO-NPs is at 310 nm.^39^ The exact wavelength of the absorption peak can be affected by factors such as particle size, shape, and synthesis method. A previous study showed the appearance of a broad absorption peak in the wavelength range of 220 nm to 400 nm.^40–42^ The results also showed additional peaks indicating the presence of residual plant extract components associated with the prepared copper oxide nanoparticles, which is consistent with previous studies.^24,43^ The UV-visible absorption spectrum showed two peaks corresponding to the cephalexin drug loaded on CuO-NPs at 230 and 300 nm, in addition to a blue shift of CuO-NPs (320 nm). Previous studies have indicated that cephalexin exhibits a higher absorption peak at 260 nm.^44^ The UV-vis spectra of CuO nanoparticles exhibited a noticeable blueshift after loading with cephalexin. The interaction of cephalexin molecules with the surface of CuO nanoparticles alters the electronic environment by redistributing charges on the CuO surface, which influences the electronic transitions between valence and conduction levels. This results in a decrease in conduction electrons and an increase in transition energy (widening the energy gap), causing the absorption to shift to a shorter wavelength (blueshift).

**Fig. 2:**
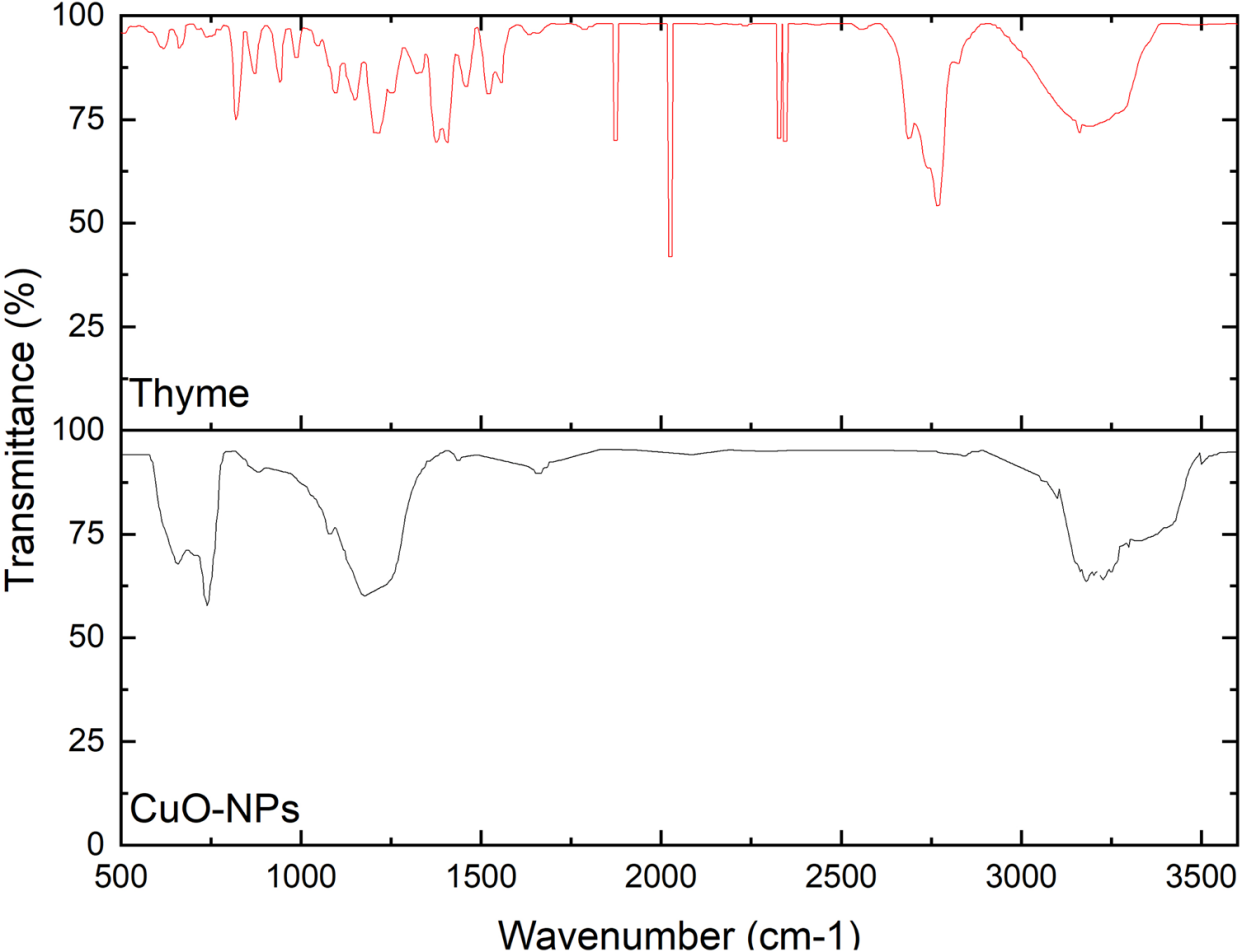
FTIR spectra of thyme extract and CuO-NPs.

**Fig. 3:**
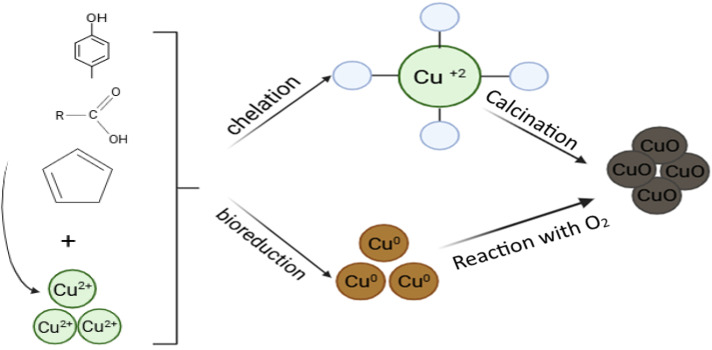
Mechanism of CuO-NP formation using thyme extract.

The FTIR spectra of thyme extract and biosynthesized CuO-NPs are shown in Fig. 2, where the spectrum of the extract shows a broad and strong peak at 3400 cm^−1^, indicating the hydroxyl groups of phenols, flavonoids, and alcohols, while in the spectrum of CuO-NPs, the same band is seen but often becomes weaker. This relative decrease indicates the participation of the hydroxyl group in the bioreduction reaction. Weak bands appear at ∼2960–2870 cm^−1^ belonging to C–H groups, which decrease after reduction, indicating a change in the chemical environment of some organic components and their encapsulation of copper ions. In addition, the spectrum of CuO-NPs shows a peak at ∼1620 cm^−1^ due to C=O of carbonyl groups, which decreases in intensity due to the interaction of carbonyl groups with copper ions. Moreover, the IR spectrum of the extract shows multiple bands in the fingerprint region (1500–1000 cm^−1^) attributed to C–O, C–C, and C=C aromatic vibrations of phenolic and flavonoid components. Their intensity decreases after the formation of CuO nanorods, indicating the involvement of hydroxyl and carbonyl groups in the reduction processes. New peaks appear in the region of ≈600–400 cm^−1^, which are due to Cu–O vibrations, confirming the formation of CuO nanoparticles. These spectral changes support the mechanism by which phytochemicals from thyme extract reduce Cu^2+^ to CuO. Fig. 3 shows the formation of copper oxide nanorods using thyme extract.

### 3.2. X-ray diffraction (XRD) analysis

**Fig. 4:**
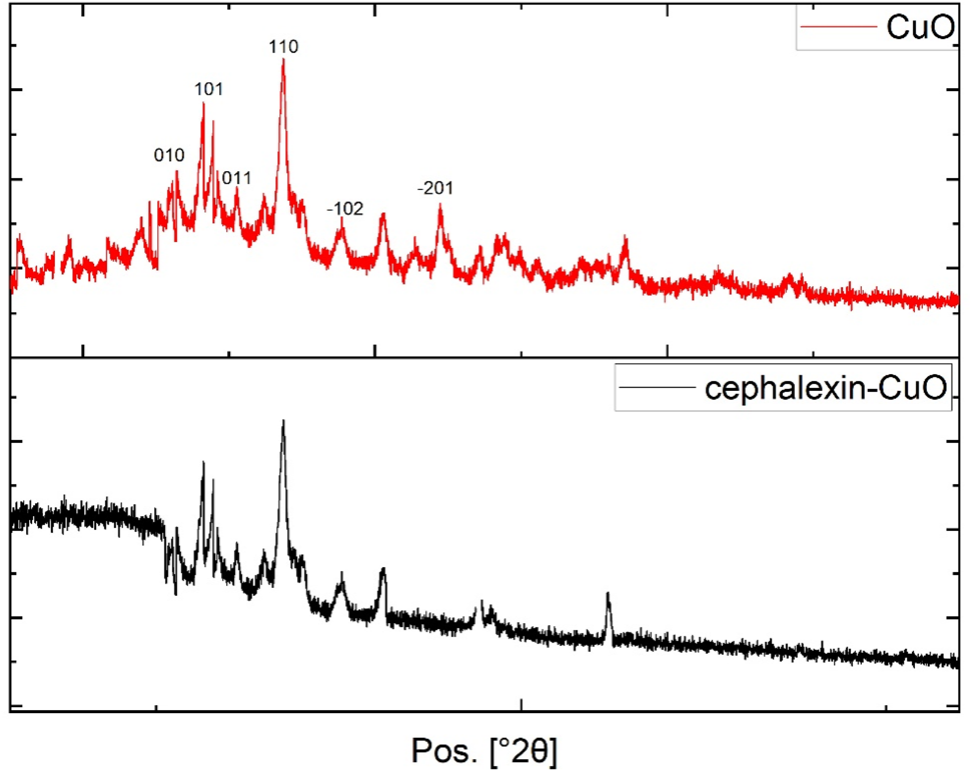
X-ray diffraction patterns of copper oxide nanoparticles and cephalexin–copper oxide nanoparticles.

X-ray diffraction analysis was used to examine the crystalline phases and crystallinity of the obtained CuO NPs. Fig. 4 shows the results of the XRD analysis, which revealed characteristic peaks indicating the presence of crystalline CuO-NPs. Miller indices (*hkl*) were assigned to the observed values by comparison with reference data. Using the Bragg equation,^45^ we calculated the distance between planes (*d*-spacing), which occurs at 2*θ* = 28.63° (*d*-spacing = 0.31 nm), 2*θ* = 33.7° (*d*-spacing = 0.26 nm), and 2*θ* = 37.6° (*d*-spacing = 0.23 nm). These peaks are consistent with JCPDS-48-1548,^46^ indicating that the CuO-NPs have a monoclinic crystal structure. These peaks also align with previous studies,^47,48^ confirming the phase purity. The lattice constants for the monoclinic system were also calculated, showing the best fit at *a* = 4.685 Å, *b* = 3.423 Å, *c* = 5.132 Å, and *β* = 99.52°. Additionally, the Scherrer equation revealed an average particle size of 8.98 nm for the synthesized copper oxide, while a previous study reported particle sizes between 16 and 18 nm.^49^ X-ray diffraction spectra demonstrated a single-phase structure of copper oxide produced *via* chemical and biological methods, with a crystal size of CuO-NPs ranging from 2.05 to 3.00 nm.^50^ Furthermore, the Williamson–Hall method was applied to estimate the strain (microstrain), where crystals were approximated to be 17.0 nm in size with a strain of 3.3 × 10^−3^. The dislocation density (*δ* = 1/*D*^2^) was found to be 5.58–3.45 × 10^15^ m^−2^, due to the numerous edges and boundaries of the nanopillars. These findings indicate that the crystals are clearly nanosized with relatively small lattice strain caused by defects and distortions, aligning with published reports for CuO samples.^51^ Therefore, structural calculations validate the formation of a nanoscale monoclinic CuO phase, with lattice constants within the expected range, and small crystal sizes supporting the desired nanoscale properties of the samples. The XRD pattern of the cephalexin–CuO compound shows a diffraction peak associated with CuO, indicating that it retains its natural crystal structure. A low-intensity peak appears at 2*θ* = 15°–25°, attributed to the drug cephalexin. These results confirm the successful preparation of the cephalexin–CuO nanocomposite.

### 3.3. Field emission scanning electron microscopy (FE-SEM) analysis

**Fig. 5:**
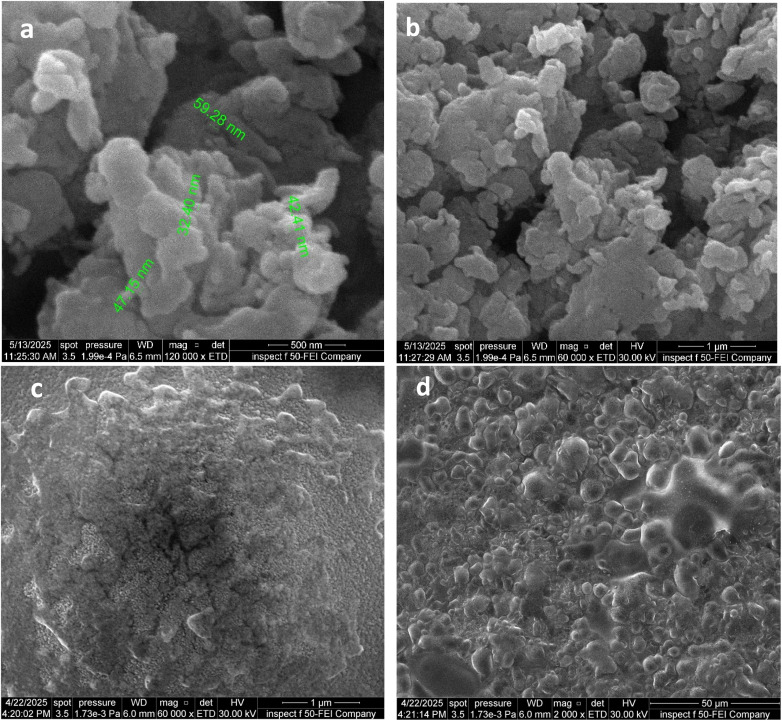
FESEM analysis of (a and b) CuO-NPs and (c and d) cephalexin–CuO nanocomposite.

Field emission scanning electron microscopy (FESEM) technology is used to determine the size and shape of prepared nanoparticles and analyze the surface properties of materials at the nanoscale. Fig. 5a and b shows images of the prepared CuO-NPs, which are semi-spherical and irregular in shape, with some agglomerates present. FESEM analysis revealed that the size of the copper oxide nanoparticles ranges from 32 to 59 nm. These results are consistent with previous studies showing the formation of spherical copper oxide particles with agglomerates and an average size of 35 nm, while Yassin reported sizes ranging from 33.5 to 63.27 nm.^52,53^ When cephalexin was loaded onto these particles, a noticeable change in the surface structure and size uniformity was observed, with sizes ranging from 13 to 31 nm, as shown in Fig. 5c and d. The presence of irregular shapes, likely due to the cephalexin drug, and particles with nanoscale sizes, likely due to the prepared copper oxide nanoparticles, indicates that the drug was successfully stabilized on the surface of the nanoparticles. There was a clear reduction in agglomeration, enhancing the possibility of using this compound in pharmaceutical applications or as an active material with improved nanoscale properties.

### 3.4. Cephalexin susceptibility test

Clinical specimens were isolated from the sputum of pneumonia patients. They were grown on MacConkey agar and blood agar under strict sterile conditions for identification. After the incubation period, the bacterial isolates were identified by examining their morphological characteristics. The colonies on blood agar were large, white, edged, and sticky owing to the presence of the capsule surrounding them, whereas the colonies on the MacConkey agar were large, convex, and pink-colored as they were able to ferment lactose. Besides morphological diagnosis, it was also diagnosed through biochemical tests as well as the utilization of the Vitek 2 system device. The results indicated that the bacterial isolates were *Klebsiella pneumonia*. After the acid-fastness test, the bacteria were then tested for cephalexin sensitivity. The results of the Vitek test indicated that *Klebsiella pneumoniae* bacteria are resistant to cephalosporin classes, of which cephalexin is an antibiotic. Furthermore, the Kirby method results indicated that after the acid-fastness test, *Klebsiella pneumonia* bacteria showed significant resistance against the cephalexin disc. The results based on the Al-Nuaimi technique revealed that *Klebsiella pneumonia* bacterial isolates were effectively resistant, as can be seen at all concentrations of cephalexin used: 10 mg ml^−1^, 20 mg ml^−1^, 30 mg ml^−1^, and 40 mg ml^−1^. The results are clear from Fig. S1.

The primary targets of antibiotics include the bacterial cell wall, where they may induce cell lysis by disrupting peptidoglycan integrity, in addition to interfering with protein synthesis, nucleic acid replication, or metabolic pathways.^54,55^ However, *Klebsiella pneumoniae* exhibits remarkable resistance mechanisms that undermine the activity of antibiotics such as cephalexin. This resistance is largely attributed to the secretion of β-lactamase enzymes, which hydrolyze the β-lactam ring and render the drug ineffective.^56^ Another major mechanism involves structural alterations in penicillin-binding proteins (PBPs), which reduce the affinity of β-lactam antibiotics such as cephalexin for their binding sites, thereby enhancing resistance.^57^ Furthermore, the ability of *K. pneumoniae* to form biofilms provides an additional protective barrier against antibiotic action.^58,59^

Previous studies, including Husna *et al.*,^60^ have demonstrated that Gram-negative bacteria, including *K. pneumoniae*, can develop resistance to cephalosporins through the production of extended-spectrum β-lactamases (ESBLs). Similarly, Li *et al.*^61^ reported that 56% of cKP strains and 17% of hvKP strains carried ESBL genes. Despite these findings, there is still limited research specifically addressing the resistance of *K. pneumoniae* to cephalexin and the underlying mechanisms that drive this resistance.

Therefore, this study aims to fill this gap by investigating the susceptibility of *K. pneumonia* to cephalexin, with a particular focus on the role of β-lactamase production, PBP alterations, and biofilm formation. By clarifying these mechanisms, the research provides new insights into the growing problem of antibiotic resistance and highlights the urgent need for effective strategies to overcome cephalexin resistance in *K. pneumoniae*.

### 3.5. Antibacterial activity assay of aqueous CuO-NPs and MIC determination

The bioactivity of CuO-NPs against *Klebsiella pneumoniae* was determined in three concentrations (2.2, 1.8, and 1.3 mg ml^−1^) to determine the minimum inhibitory concentration (MIC), as shown in Fig. S2. The results revealed maximum inhibition of bacterial growth at 2.2 and 1.8 mg ml^−1^ with high antibacterial activity, whereas partial bacterial growth was observed at 1.3 mg ml^−1^. This confirms that the MIC will be between 1.3 and 1.8 mg ml^−1^. The greater activity of CuO-NPs is rationalized by their ability to penetrate into the cell wall of bacteria, destabilize vital cellular components, and maintain effective dispersion without aggregation.^50,62^ The reduced growth at low levels of concentration presents an early-stage inhibitory effect, which is indicative of the dose–response relationship.^63,64^

**Fig. 6:**
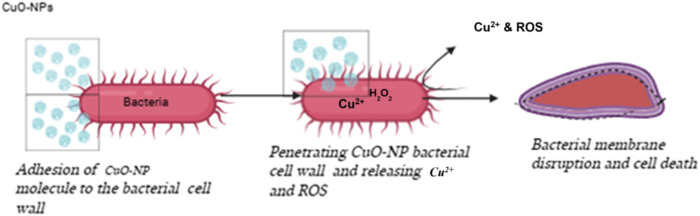
Mechanism of CuO-NPs as antibacterial activity and release of ROS against *K. pneumoniae*.

Notably, the findings in this study demonstrate the potential of CuO-NPs in avoiding bacterial resistance processes in *K. pneumoniae*. By causing oxidative stress and damage to critical cellular components, as shown in Fig. 6, CuO-NPs can suppress growth even in highly resistant strains of the conventional antibiotics. This supports Rábago *et al.*,^65^ who reported enhanced production of ROS in some strains of bacteria with greater concentrations of CuO-NPs, although this was not total at lower concentrations. In contrast, Nagore *et al.*^66^ noted weaker antibacterial activity, highlighting the heterogeneity of bacterial susceptibility. Worldwide, the current research testifies to the promise of CuO-NPs as potent agents against antibiotic resistance in *K. pneumoniae*.

### 3.6. Antibacterial activity assay of aqueous cephalexin–CuO-NPs

In the present study, the cephalexin–CuO-NP compound exhibited potent inhibitory activity against *Klebsiella pneumoniae*, where the growth was completely inhibited at a concentration of 30 mg ml^−1^, whereas the other concentrations (10, 20, and 40 mg ml^−1^) tested did not reveal the same level of efficacy (Table 1 and Fig. S3). The results point towards the prospects of metallic nanoparticles, especially in conjunction with antibiotics, to combat bacterial resistance. While nanoparticles alone are toxic, the conjugation of antibiotics with nanoparticles enhances antimicrobial activity, reduces dose, and reduces toxicity overall.

**Table 1:** Antibacterial activity of different concentrations of cephalexin, CuO NPs, and cephalexin–CuO nanocomposite against *K. pneumoniae*

Cephalexin		CuO NPs		CuO–cephalexin nanocomposite	
10 mg ml^−1^	Non inhibition	1.3 mg ml^−1^	Semi-inhibition	10 mg ml^−1^	Non inhibition
20 mg ml^−1^	Non inhibition	1.8 mg ml^−1^	Complete inhibition	20 mg ml^−1^	Non inhibition
30 mg ml^−1^	Non inhibition	2.2 mg ml^−1^	Complete inhibition	30 mg ml^−1^	Complete inhibition

Activity at 30 mg ml^−1^ suggests that this is the concentration where the adsorption of cephalexin molecules on the surface of CuO-NPs is optimal, and there is a synergistic effect that allows for deeper penetration of the bacterial cell wall and inhibits essential cellular processes.^65,66^ At lower concentration values (10 and 20 mg ml^−1^), the number of antibiotic molecules bound to nanoparticles appeared insufficient to determine effective inhibition, while at 40 mg ml^−1^, nanoparticle aggregation likely reduced availability on the surface and antimicrobial activity.^67^ Such results imply that CuO-NPs have the ability to protect cephalexin from β-lactamase enzymes of bacteria and increase its binding on target sites, hence preventing resistance mechanisms within *K. pneumoniae*. Such a synergistic function supports previous reports by Zhang *et al.*,^68^ in which antibiotic–nanoparticle conjugates exhibited superior activity against Gram-positive and Gram-negative bacteria, including multidrug-resistant bacteria.

### 3.7. Antioxidant efficiency

**Fig. 7:**
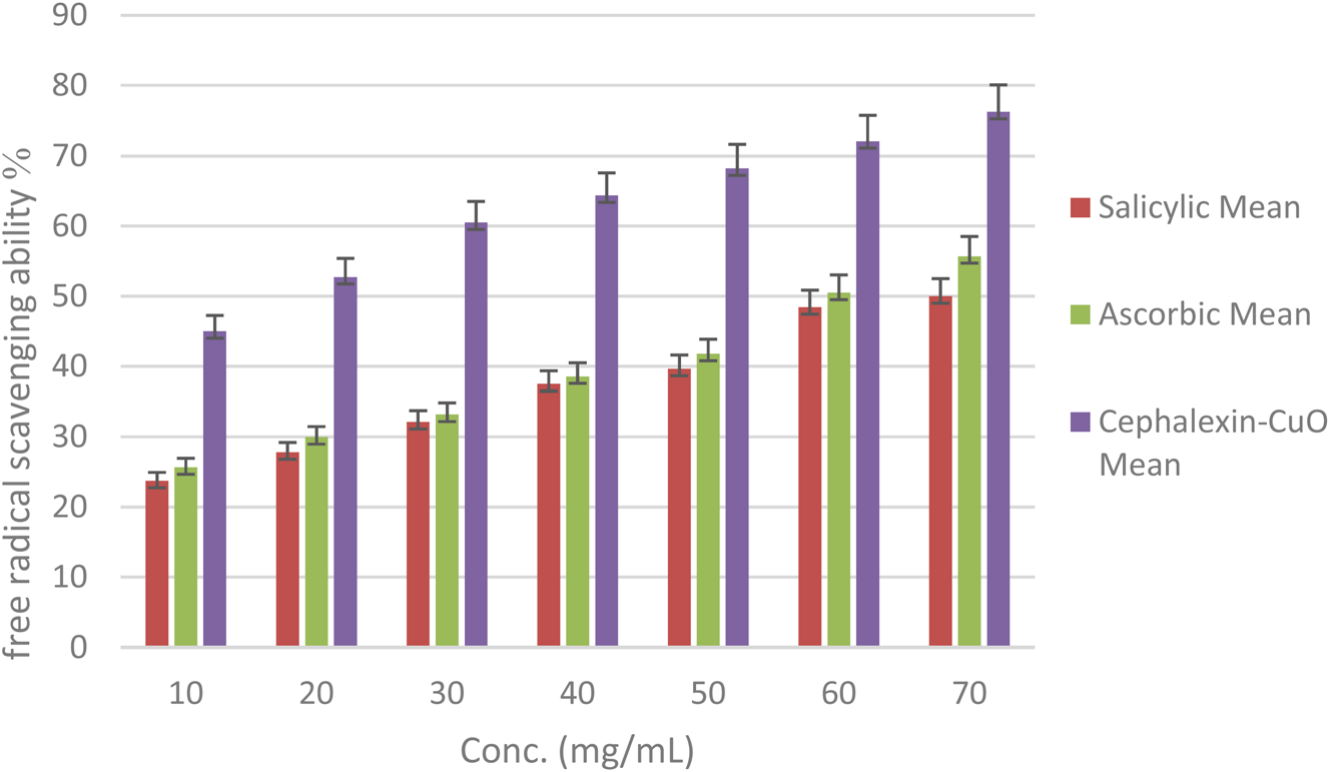
Evaluation of the free radical scavenging ability of the DPPH compound using the cephalexin–CuO nanocomposite, ascorbic acid, and salicylic acid.

The ability of the cephalexin–CuO nanocomposite to remove free radicals was studied to verify its antioxidant properties. One of the most important applications of composite nanomaterials is their antioxidant activity, which plays a vital role in all living systems. The ability of the hybrid compound to remove free radicals generated by DPPH was measured and compared to that of some standard compounds. As shown in Fig. 7 and Table 2, the current results revealed high antioxidant activity in a concentration-dependent manner. Ascorbic acid and salicylic acid were used as standard reference materials. The cephalexin–CuO nanocomposite exhibited the greatest DPPH free radical scavenging activity at concentrations ranging from 10 to 70 mg ml^−1^. The standard DPPH density is reduced by accepting hydrogen or electrons.^50^ Decreases in sample activity were measured by changes in DPPH color, with the highest removal rate (75.9%) observed, while ascorbic acid and salicylic acid exhibited removal rates of 55.7% and 50.3%, respectively, under the same conditions, indicating the prepared compound's higher antioxidant activity. A very low concentration of the cephalexin–CuO nanocomposite compound eliminated 45% of free radicals. Therefore, the cephalexin–CuO nanocomposite's removal efficiency is much higher than that of the standard compounds. These results suggest that the bio-nanocomposite shows promise as a new antioxidant agent.

**Table 2:** Evaluation of the free radical scavenging ability percentage of the DPPH compound using the cephalexin–CuO nanocomposite, ascorbic acid, and salicylic acid

Conc. (mg ml^−1^)	Salicylic acid, mean ± SD	Ascorbic acid, mean ± SD	Cephalexin–CuO, mean ± SD	*P*-value
10	23.74 ± 1.19	25.67 ± 1.28	45.00 ± 2.25	<0.005
20	27.82 ± 1.39	29.97 ± 1.50	52.75 ± 2.64	
30	32.12 ± 1.61	33.19 ± 1.66	60.50 ± 3.02	
40	37.49 ± 1.87	38.56 ± 1.93	64.37 ± 3.22	
50	39.63 ± 1.98	41.78 ± 2.09	68.24 ± 3.41	
60	48.43 ± 2.42	50.51 ± 2.53	72.12 ± 3.61	
70	50.00 ± 2.50	55.71 ± 2.79	76.24 ± 3.81	

### 3.8. Biofilm inhibition assay

**Fig. 8:**
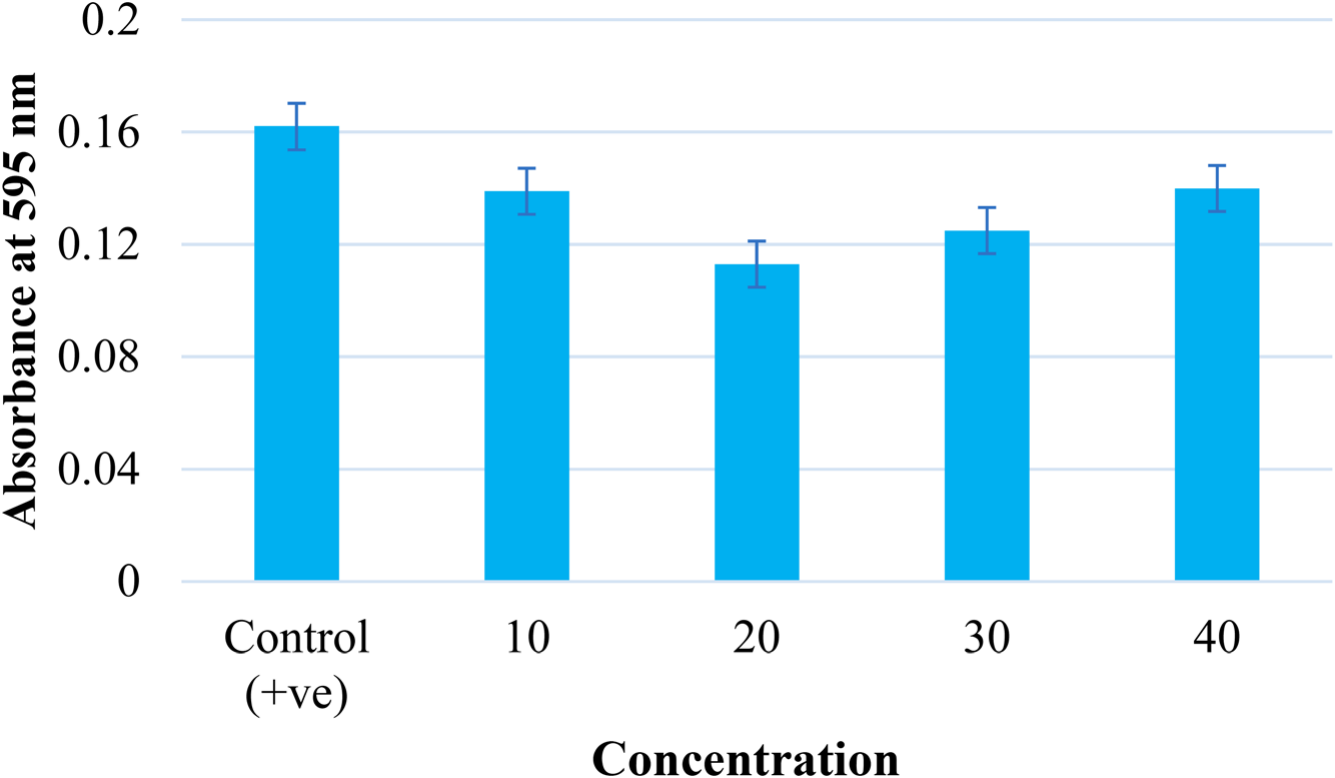
Evaluation of the impact of the cephalexin–CuO nanocomposite on the inhibition of *Klebsiella pneumoniae* biofilms.

*Klebsiella pneumoniae* biofilm formation was significantly inhibited by the cephalexin–CuO nanocomposite, as measured by biofilm biomass at 595 nm. Fig. 8 shows the quantitative assessment with mean absorbance values and standard error bars. Compared to the positive control (0.162 ± 0.006), 20 mg ml^−1^ treatment caused the most significant inhibition (0.113 ± 0.004, *p* < 0.01). Interestingly, this effect was greater than that observed at greater concentrations (30 mg ml^−1^ = 0.130 ± 0.005 and 40 mg ml^−1^ = 0.140 ± 0.006), suggesting that overdosing can trigger defense mechanisms in bacteria. At 10 mg ml^−1^, a small reduction was also observed (0.139 ± 0.005).

These findings indicate that the optimum concentration is 20 mg ml^−1^, maybe due to receptor saturation at this concentration, beyond which further increases in concentration trigger extracellular polymeric substance (EPS) production and inhibit antibiotic penetration. This impact was also observed by Grandière-Pérez *et al.*,^69^ who reported enhanced β-lactamase production at high cephalosporin concentrations, and by Bai *et al.*,^70^ who noted the higher efficacy of lower CuO nanoparticle concentrations in biofilm disruption.

### 3.9. Cytotoxicity test

The MTT assay was used to assess the cytotoxicity of the produced CuO-NPs on Baby Hamster Kidney (BHK-21) cell lines. The results demonstrated that at the examined concentrations (10–40 μg ml^−1^), CuO-NPs were not cytotoxic. In comparison to 100 ± 1.5% for untreated control cells, cell viability was determined to be 96 ± 2.1% at 10 μg ml^−1^, 94 ± 1.8% at 20 μg ml^−1^, 92 ± 2.4% at 30 μg ml^−1^, 90 ± 2.8%, and 88 ± 2.7% at 40 μg ml^−1^. Doxorubicin, the positive control, confirmed the sensitivity by exhibiting the anticipated significant reduction in cell viability (38 ± 2.3%), verifying the assay's biocompatibility. These results demonstrate CuO-NPs' high biocompatibility with healthy mammalian cells, confirming their potential for use in biomedical applications at the concentrations used. Even at a concentration of 40 μg ml^−1^, green-synthesized CuO-NPs show good cell survival and minimal toxicity, according to the results of cytotoxicity assays. This implies their use in pharmaceutical and biological applications, specifically in the delivery of drugs and the treatment of microorganisms. The significant cell viability suggests that the nanoparticles are biocompatible with healthy mammalian cells and provide little risk of negative side effects when used in a medicinal setting. The green manufacturing technique, which uses phytochemicals from plant extracts as natural capping agents and stabilizers to improve nanoparticle biocompatibility and suppress host cell oxidative stress, is responsible for the decreased cytotoxicity seen in this study.^71^ N. Mahmoud *et al.*^72^ found that biosynthesized CuO-NPs were less cytotoxic than chemically produced ones. Furthermore, Hassan *et al.*^73^ confirmed the current study's findings by highlighting the therapeutic utility of biogenic CuO-NPs as safe and efficient antibacterial agents.

Overall, these findings support the low cytotoxicity of CuO-NPs produced with environmentally friendly techniques, offering a secure foundation for further biomedical research, including the use of these particles in formulations loaded with cephalexin to boost antibacterial activity.
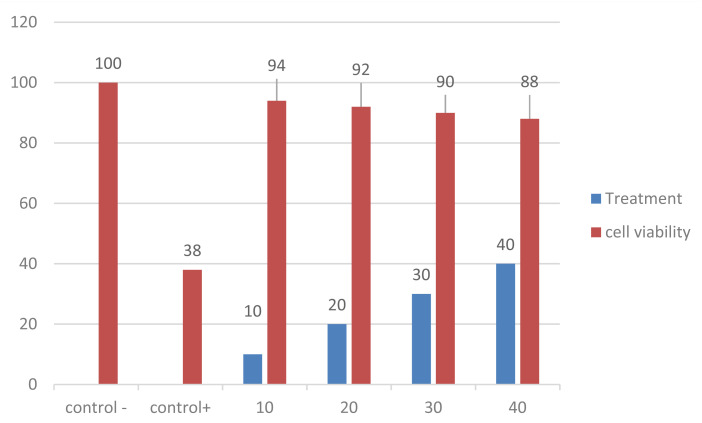


## 4. Conclusion

The current study aimed to optimize the biological properties of cephalexin by loading it onto copper oxide nanoparticles prepared by a green method using wild thyme extract. The nanoparticles were screened using different techniques and proved to be biologically active against *Klebsiella* isolated from patients with pneumonia at an optimal CuO nanoparticle concentration of 1.8–2.2 mg ml^−1^. The cephalexin–copper oxide nanocomposite achieved complete inhibition of the bacteria at 30 mg ml^−1^ and showed the ability to reduce biofilm formation at 20 mg ml^−1^, in addition to antioxidant activity. These results demonstrate that the prepared nanocomposite represents a promising approach for the development of novel antibacterial and antioxidant therapeutic agents, with future potential for clinical applications.

## Ethical statement

We declare that all experiments and procedures described in this study were conducted in accordance with the relevant national and international laws and institutional guidelines of the University of Anbar.

All experimental protocols were reviewed and approved by the Institutional Ethics Committee/Animal Care Committee of the University of Anbar.

Informed written consent was obtained from all participants prior to sample collection, following the principles of the Declaration of Helsinki.

## Author contributions

All authors contributed to the preparation, conceptualization, design, data collection, and analysis of the study's material and approved the final version of the publication.

## Conflicts of interest

The authors affirm that they have no relevant financial interests or personal relationships that could have influenced the work presented in this paper.

## Supplementary Material

NA-OLF-D5NA00726G-s001

## Data Availability

Data will be made available upon request. Supplementary information is available. See DOI: https://doi.org/10.1039/d5na00726g.
