# Supplementary material for: Green synthesis of CuO nanoparticles using thyme extract and their application as cephalexin carriers against Klebsiella pneumoniae
Source: Nanoscale Adv. 2025 Nov 11;8(1):97–110. doi: 10.1039/d5na00726g (PMC12604053; doi:10.1039/d5na00726g)
Supplement: NA-008-D5NA00726G-s001 [file NA-008-D5NA00726G-s001.pdf]

Supplementary Information (SI) for Nanoscale Advances.
This journal is © The Royal Society of Chemistry 2025

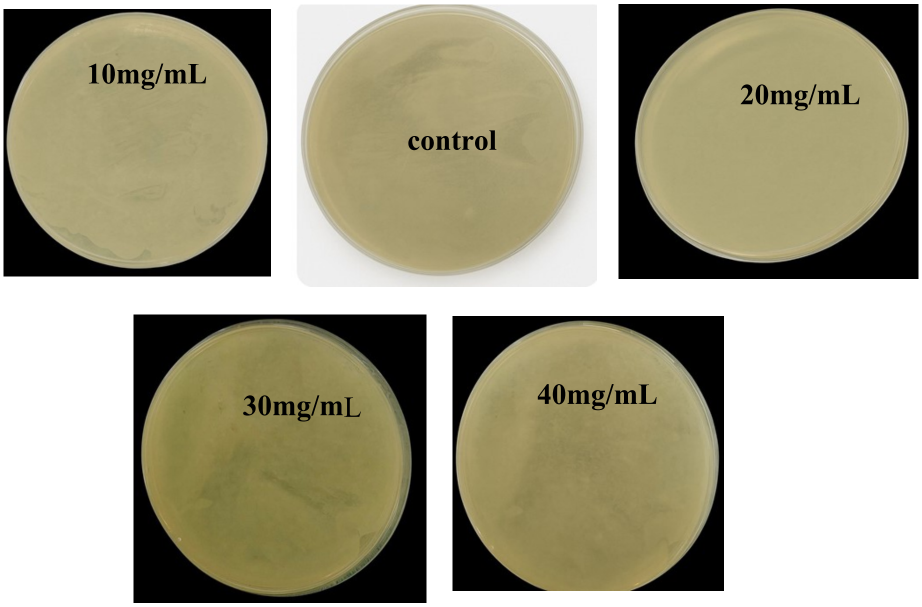

. S1: Effective resistance of *Klebsiella pneumoniae* to cephalexin at different concentrations

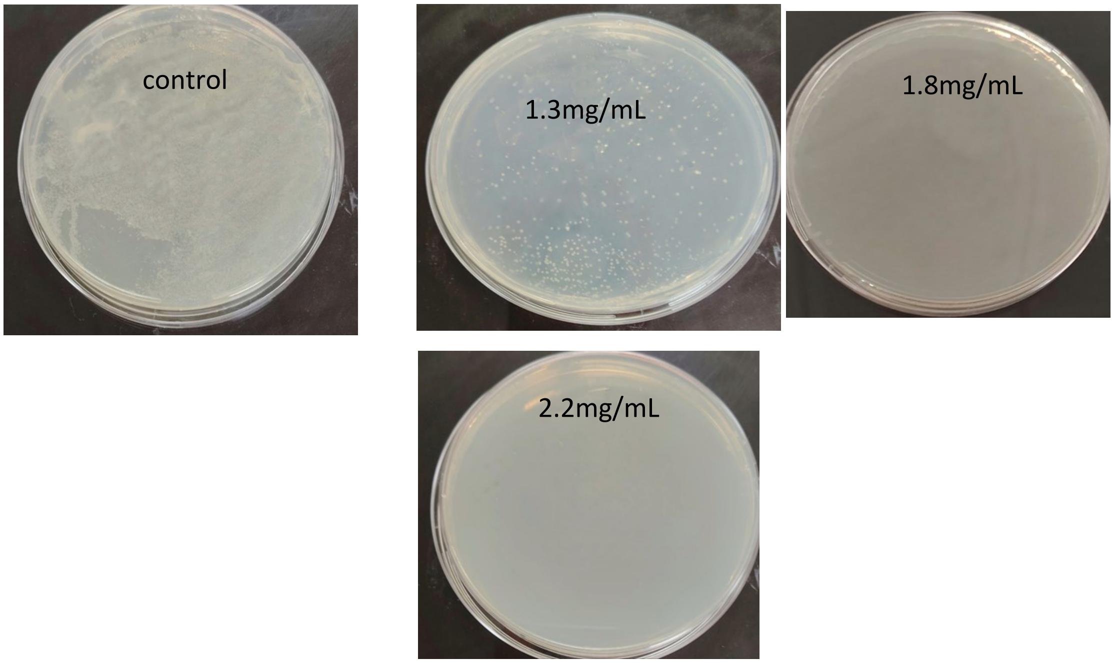

S2: Antibacterial activity of different concentration of aqueous CuO-NPs
